# Effects on 24-hour blood pressure variability of ace-inhibition and calcium channel blockade as monotherapy or in combination

**DOI:** 10.1038/s41598-018-31746-2

**Published:** 2018-09-13

**Authors:** Gianfranco Parati, Paolo Castiglioni, Stefano Omboni, Andrea Faini

**Affiliations:** 1 Department of Medicine and Surgery, University of Milano-Bicocca, 20126 Milan, Italy; 2 Istituto Auxologico Italiano, IRCCS, Department of Cardiovascular Neural and Metabolic Sciences, S. Luca Hospital, Milan, 20149 Italy; 3 IRCCS Fondazione Don Carlo Gnocchi, Milan, 20148 Italy; 4 Italian Institute of Telemedicine, Solbiate Arno (VA), Varese, 21048 Italy; 5 Scientific Research Department of Cardiology, Science and Technology Park for Biomedicine, Sechenov First Moscow State Medical University, Moscow, 119992 Russian Federation

## Abstract

Cardiovascular events in hypertensives are associated with elevated average blood pressure (BP) and higher short-term BP variability (V), but little is known on treatment effects on BPV and on how to assess changes in short-term BPV. Aim of our study was to address the methodology of short-term BPV assessment and its reduction by Lercanidipine (L) or Enalapril (E) and their combination, through analysis of 24-hour ambulatory BP recordings from two studies including subjects of different age. Study-1: 64 middle-age hypertensives (52.9 ± 9.5 yrs) received L and E s.i.d. at 10 mg (L10, E10) or 20 mg doses (L20, E20) for 8 weeks. Study-2: 66 elderly hypertensives (65.5 ± 4.7 yrs) received placebo, L10, E20 and L10 + E20 s.i.d. for 4 weeks. In middle-age subjects, both L and E decreased mean BP and, at the highest dose, also short-term BPV. In elderly subjects, L10 alone or in combination with E20 reduced BPV. Treatment-induced reductions in BP levels and BPV were uncorrelated. Different methods for short-term BPV assessment did not always provide superimposable results in the elderly. Our study supports a better reduction of BPV by L in the elderly and by E + L combination at any age, suggesting BPV reduction to be independent from reduction in average BP.

## Introduction

Evidence is available from observational studies and trials meta-analyses that cardiovascular events in hypertensive patients are associated not only with elevated average blood pressure (BP) levels but also with increased BP variability (V), in particular with an increased short-term BPV over 24 hours. 24 h BPV is a complex phenomenon, including BP fluctuations at different frequency, ranging from day-night BP changes to reading–to-reading BP variations, the former being associated with favorable, and the latter with unfavourable effects on organ damage and prognosis in hypertensive patients^1^. Thus, when quantifying BPV over 24 hours, it has been recommended to separately assess the degree of day-night BP reduction (dipping), whose occurrence is associated with a favorable impact on organ damage and prognosis, and the magnitude of faster BP fluctuations (short-term BPV), an increase of which is on the contrary associated with increased severity of organ damage and higher rate of cardiovascular events and cardiovascular mortality^1^. For this reason, recent methods for estimating 24 hour BPV have been proposed which allow assessment of short-term BPV while excluding the contribution of day-night BP changes^1,2^. Furthermore, on such a background, it has been suggested that anti-hypertensive treatment should be aimed at reducing not only BP levels, but also short-term BPV, while preserving nocturnal BP dipping^1^. The actual ability of antihypertensive treatment to reduce short-term BPV is still a matter of debate, however, as previous studies suggested that a reduction in BP standard deviation by treatment could be only the consequence of concomitant reduction in BP mean, without evidence of any direct and specific effect by antihypertensive drugs on BPV^3^. Moreover, limited information is available also on whether there is any difference among drug classes in this regard.

**Figure 1:**
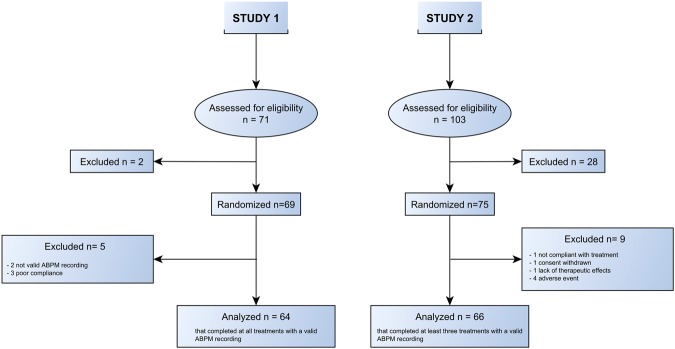
Study flowchart and CONSORT diagram.

Therefore, aim of this work was to address the methods for assessing short-term 24 h BPV and to explore the effects on short-term BPV, measured over 24 hours by ambulatory BP monitoring (ABPM), of monotherapy with two commonly used antihypertensive drugs, targeting different mechanisms of cardiovascular regulation, i.e., a dihydropyridine calcium channel blocker (CCB) and an angiotensin converting enzyme inhibitor (ACEI), as well as of their combination. This was done by re-analyzing ABPM recordings, obtained in two previous studies aimed at exploring the 24-hour antihypertensive effect of the CCB Lercanidipine (L), the ACEI Enalapril (E) and their combination in essentially hypertensive patients of different ages: in middle-age subjects and in elderly subjects (Fig. 1).

## Results

### Middle-age patients (study 1)

**Figure 2:**
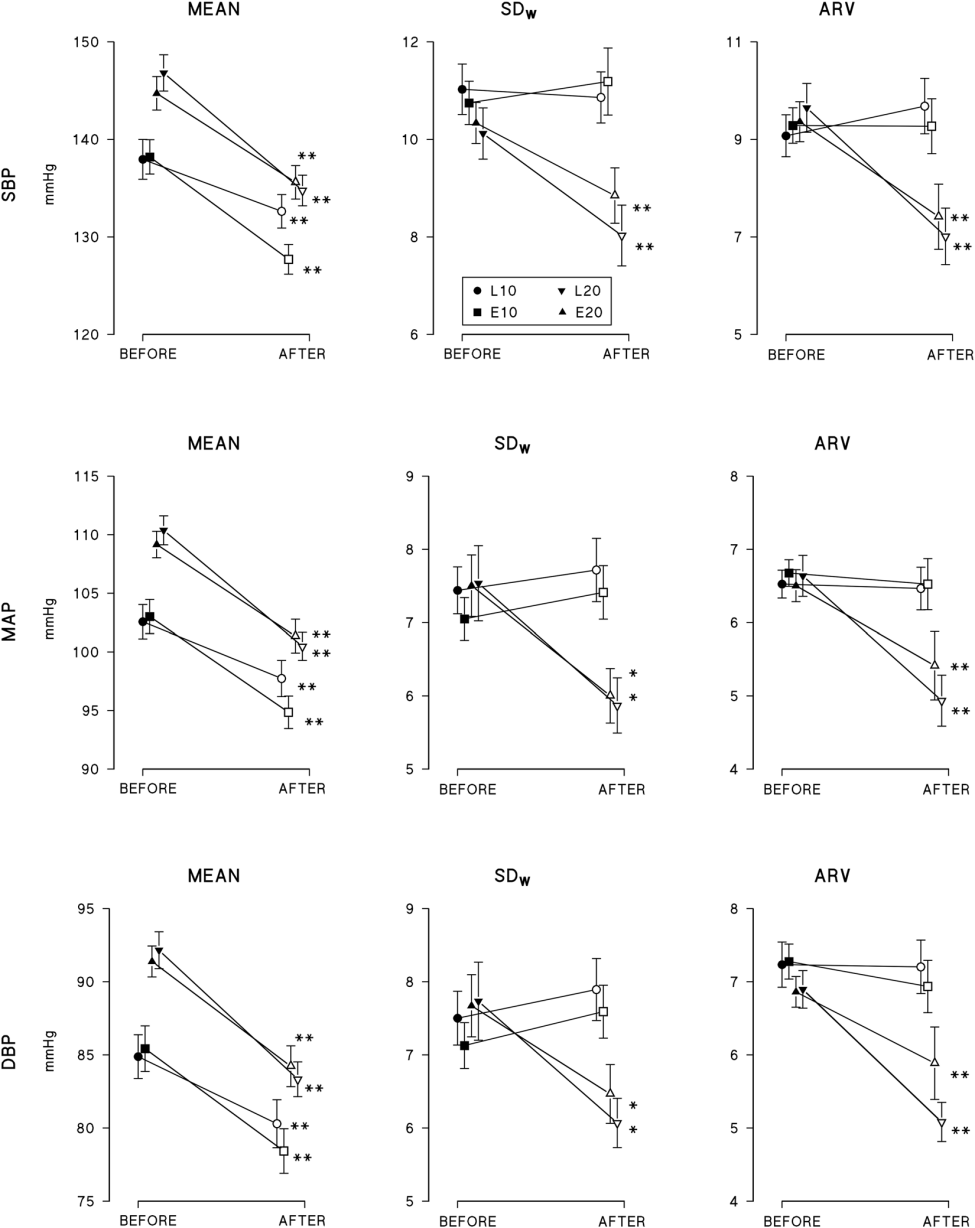
BP levels and indices of short-term BP variability (SD_W_ and ARV) over 24 hours in middle-age hypertensives (Study 1) as mean and standard error of the mean before (full symbols) and after (open symbols) each of four treatments (circle: L10; square: E 10; downward arrow: L20; upward arrow: E 20); the *and **indicate differences after vs. before treatment significant at 0.05 and 0.01 statistical levels. See text for abbreviations.

Figure 2 shows the effects of treatments with L and E at 10 mg (L10, E10) and 20 mg (L20, E20) dosages separately on Systolic (S)BP, Mean Arterial Pressure (MAP) and Diastolic (D)BP mean levels and on two short-term BPV indices: the weighted standard deviation (SD_W_) and the average real variability (ARV). All treatments decreased significantly BP means over 24 hours. Short-term BPV indices were not influenced by low dose treatments, neither L10 nor E10, while high-dose treatments, L20 and E20, decreased significantly both indices. Reductions of short-term variability for MAP and DBP were more significant when quantified by ARV than by SD_W_.

Before treatment, patients who received 20 mg doses (L20 and E20) had higher BP than those receiving 10 mg doses: this is likely due to a bias introduced by dose adjustment from 10 mg to 20 mg for those hypertensive individuals that did not normalize DBP after one month of the low dose treatment (see methods). However, the dose-adjustment procedure did not affect short-term BPV indices, being baseline values of ARV or of SD_W_ similar for all treatments.

No significant associations were found between decreases in 24 hour levels of SBP, MAP or DBP, and decreases in their corresponding weighted standard deviation (SD_W_) for any of the four treatments (Table 1), with the exception of the effect of the E20 treatment on SBP. This association, however, was characterized by a negative slope which means that larger decreases in mean level were not associated with larger, but with smaller decreases of SD_W_.

**Table 1:** Linear regression analysis between decrease in BP mean level and decrease in short-term BPV, as quantified by ΔSD_W_.

DBP		slope	R^2^	explained	P
*Study 1: after vs*. *before treatment*	L10	0.48	0.028	1.4%	0.40
	E10	−1.29	0.102	5.2%	0.09
	L20	0.60	0.070	3.6%	0.22
	E20	0.07	0.001	0.1%	0.87
*Study 2: treatment vs*. *placebo*	L10	0.36	0.013	0.7%	0.36
	E20	0.10	<0.001	<0.1%	0.81
	L10 + E20	0.24	0.003	0.2%	0.66
**MAP**					
*Study 1: after vs*. *before treatment*	L10	0.43	0.020	1.0%	0.48
	E10	−1.36	0.109	5.6%	0.08
	L20	−0.52	0.045	2.3%	0.40
	E20	0.39	0.025	1.3%	0.48
*Study 2: treatment vs*. *placebo*	L10	0.72	0.030	1.5%	0.17
	E20	0.32	0.007	0.4%	0.51
	L10 + E20	0.63	0.023	1.2%	0.23
**SBP**					
*Study 1: after vs*. *before treatment*	L10	0.79	0.062	3.1%	0.21
	E10	−0.34	0.017	0.9%	0.49
	L20	0.015	0.005	0.3%	0.76
	E20	−2.11	0.540	32.3%	<0.001
*Study 2: treatment vs*. *placebo*	L10	1.32	0.055	2.8%	0.06
	E20	0.31	0.004	0.2%	0.60
	L10 + E20	1.28	0.089	4.6%	0.02

R^2^ (=determination coefficient) and slope estimated from regression model; explained = fraction explained by the model (i.e., percentage of SD_W_ changes correlated with changes in mean level).

### Elderly patients (study 2)

**Figure 3:**
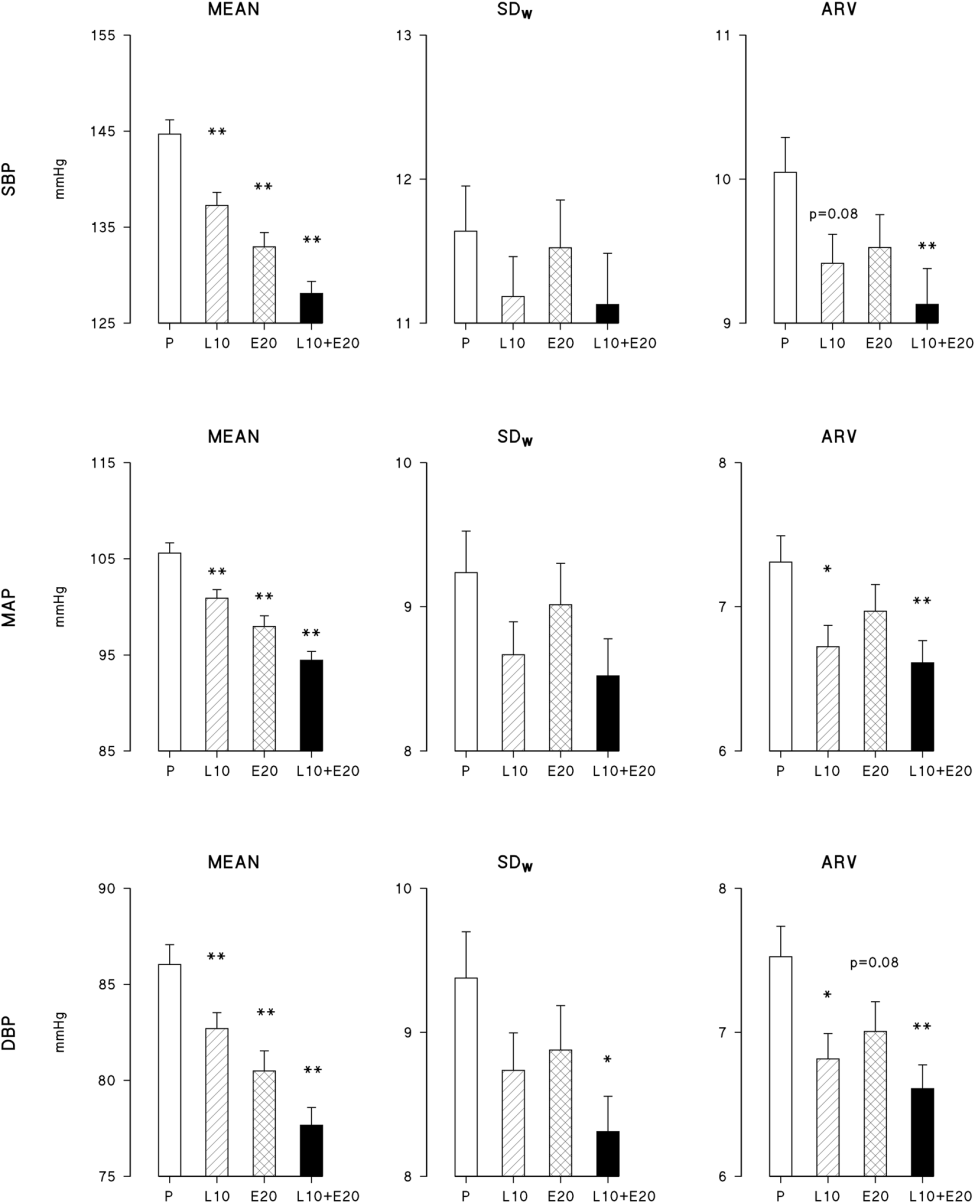
BP levels and indices of short-term BP variability over 24 hours (SD_W_ and ARV) in elderly hypertensives (study 2) for each of four treatments (mean and standard error of the mean); the *and **indicate differences vs. placebo (P) significant at 0.05 and 0.01 statistical levels. See text for abbreviations.

Monotherapies with active compounds as well as their combination significantly decreased the 24-hour BP means compared to placebo in elderly hypertensive patients (Fig. 3). The decrease was more pronounced for E20 than for L10 (p < 0.01) and for the combined treatment L10 + E20 than for E20 (p < 0.01).

Considering monotherapies, L10 decreased ARV significantly for MAP and DBP, while E20 did not have significant effects on short-term BPV. The combined treatment, L10 + E20 had marked effects on ARV, which decreased significantly for SBP, DBP and MAP. The combination treatment decreased also SD_W_, but for DBP only.

No significant associations were found between changes vs. placebo in 24-hour mean levels and changes vs. placebo in SD_W_ of MAP and DBP for any active treatment (Table 1). As to SBP, a significant positive relationship between decreases in SBP level and decreases in SD_W_ of SBP was found for the L10 + E20 treatment only. However, the fraction of change in SD_W_ explained by the linear model was less than 5%.

## Discussion

On the background of the accumulating evidence that cardiovascular events in hypertensive patients are associated not only with higher BP mean levels but also with increased BPV, in particular when focusing on short-term BPV over 24 hours^1^, our study contributes to the debate on the possibility that antihypertensive treatments have a direct effect in buffering BPV, by investigating a few issues of practical relevance. In particular, our study addresses three aspects: (1) the possible difference in the effects on BPV exerted by two classes of drugs (ACEI and CCB) and by their combination; (2) the possible influence of ageing on the effects of these drugs on BPV; and (3) whether a drug-induced reduction in BP standard deviation might be consequence of the concomitant drug-induced reduction in mean BP level, or whether it might rather reflect a specific drug action on BPV. To address the first two issues, we have compared the effects of an ACEI, of a CCB and of their combination on short-term BPV over 24 hours, by considering different drug dosages and their administration to individuals of different age. To address the third issue, in both studies we have investigated whether the degree of reduction in mean is or is not correlated to the degree of reduction in standard deviation.

Results of study 1 show that in middle age individuals both the CCB Lercanidipine and the ACEI Enalapril are effective in reducing 24-hour BP levels either at the dose of 10 and of 20 mg given once a day. They are also effective in reducing short-term BPV, but only at their higher dose of 20 mg. These findings suggest that low dose treatments, capable of reducing 24-hour BP levels, might not be powerful enough to also reduce BPV, i.e. the dynamic component of BP reflecting the interaction between environmental stimuli and the cardiovascular control mechanisms of a given subject.

Results of study 2, on elderly hypertensive patients, partly differ in terms of BPV from those of study 1 carried out in middle aged individuals. In fact, no reduction in short-term BPV could be seen under treatment with ACEI Enalapril 20 mg, while short-term variability of DBP and MAP as quantified by ARV were significantly reduced by CCB Lercanidipine even at the low dose of 10 mg. These results could be partly explained by the tendency towards a reduced efficacy of ACE inhibitors in elderly individuals^4,5^, in whom these drugs may be effective enough in reducing mean BP levels^6^, but may be less effective than CCBs in buffering short-term BPV. Even if the relatively low dose of CCB Lercanidipine tended to decrease ARV also for SBP, the reduction did not reach the statistical significance as for MAP and DBP. This may be due to the fact that DBP fluctuations reflect modulations of peripheral resistance better than SBP fluctuations. Therefore, they can be more effectively reduced by a CCB than by an ACEI in the elderly, in whom baroreflex modulation of peripheral resistance is known to be impaired^7^ and arterial stiffness increased. Combination treatment with an ACEI and a CCB was more effective not only in reducing 24-hour BP levels, but also in reducing most indices of BPV in this elderly cohort.

To evaluate whether an association exists between the reduction in BP level and the reduction in BP variability, we calculated a total of 21 regressions between BP mean and SD_W_ (Table 1). The only significant association with a positive slope regards the effect of L10 + E20 vs. placebo on SBP. However, the percentage of explained variability is less than 5%, indicating that even if the association were real (and not due to chance) the possible direct effect of the reduction of SBP mean on the reduction of SBP SD_W_ is minimal. This excludes that a reduction in BPV is just a consequence of the concomitant reduction in BP level due to a relation between central value and dispersion for the statistical distribution of BP measures. This lack of correlation makes also unlikely that treatments may have activated one single mechanism which deterministically reduced the BP level and the BP variability simultaneously. It may also explain why SD_W_ and ARV from 24-hour ambulatory BP monitoring were associated with highly significant hazard ratios for total mortality even after adjustment for the 24-hour BP level, which is expected to have a greater prognostic value^8^. Furthermore, it may explain the significant risk reclassification of BP variability (particularly during nighttime) when added to fully-adjusted models^9^.

A merit of our study is the assessment of 24-hour BPV with indices that specifically quantify short-term BP fluctuations (reported to carry an increased risk of cardiovascular complications), while excluding the contribution to 24-hour BPV of nocturnal BP fall (which in contrast is known to have a favourable prognostic meaning)^1,10^. In this regard, we employed two indices that quantify the effects of treatments on BPV with a different sensitivity, and this is probably due to the different weight that slower and faster components of short-term BPV have in determining ARV and SD_W_ values (see methods). To the best of our knowledge, our study is the first addressing these methodological issues in a clinical perspective, offering indications for a better standardization of methods for assessing short-term BPV in clinical trials. This is an important issue, given that proper comparison of data from previous studies exploring the clinical relevance of BPV has been so far made difficult by the diverse methods used by different investigators^1^. Therefore, our study also underlines the utility to decompose short-term BPV into faster and slower components, in order to more precisely evaluate the effects of treatments or to better stratify the cardiovascular risk associated to short-term BPV.

Our study offers information also on the possibility that some antihypertensive agents might reduce BPV independently from a reduction in mean BP levels, which is yet a debated issue^3^. The demonstration of uncorrelated changes in BP mean and standard deviation supports the usefulness of focusing on the reduction of both BP and BPV. Indeed, our results should be interpreted on the background of previous studies on this issue. In rats, BPV is an independent determinant of end-organ damage^11^ and CCB may reduce BPV and protect against end-organ damage, independently from the reduction in mean BP^12^. Also in a clinical setting CCB treatment reduced daytime and nighttime variability of SBP and improved arterial baroreflex modulation of heart rate in diabetic hypertensive patients, although this demonstration was provided through BP recordings at the finger artery level, where pulse waveforms may be differently amplified in different individuals, as a function of differences in stroke volume and arterial stiffness^13^. A more recent study evaluated the antihypertensive effects of an ACEI alone or associated with CCB or with a diuretic, and showed that BPV decreased when the ACEI was combined with a CCB, and that conversely it did not change when ACEI was combined with a diuretic^14^.

Evidence that CCBs might be superior to other drug classes in reducing BPV, probably because of their effects on arterial wall properties^1^, has also been provided by studies focusing on mid-term (day-by-day) and long-term (visit-to-visit) BPV. In particular, in a study by Matsui *et al*., mid-term BPV was quantified from day-by-day changes of home BP in patients initially treated with an angiotensin antagonist (olmesartan) and then randomly assigned to receive additionally either a CCB (azelnidipine) or a diuretic (hydrochlorothiazide)^14^. The addition of azelnidipine led to a significantly larger reduction in mid-term BPV than the addition of hydrochlorothiazide, with similar reductions in mean BP in both groups. Moreover, the BPV reduction in the azelnidipine group was independently related to a reduction in aortic stiffness (assessed through carotid-femoral pulse wave velocity)^14,15^.

Moreover, retrospective analyses of the ASCOT BPL and the UK-TIA trials focusing on visit-to-visit BPV provided evidence of a superior reduction of BPV by the CCB amlodipine as compared to other drug classes, in particular to the beta blocker Atenolol^16–18^. More recently, when focusing on 24-hour BPV in the X-CELLENT study, treatment with amlodipine, a long lasting CCB, was associated with a greater reduction in different measures of 24-hour BPV than treatment with Indapamide, Candesartan and placebo^19^. Our study offers novel information on the role of dihydropyridine CCBs in reducing short-term BPV by comparing different doses, with and without combined administration of a drug blocking the renin-angiotensin system, in patients of different age.

We should acknowledge some limitations of our work. First, it is based on the retrospective analysis of studies previously performed for other purposes, although the methodology used for ABPM did allow for an accurate assessment of parameters measuring BPV. Moreover, the number of patients investigated was not high, limitation, however, counteracted by the crossover design of these studies. Therefore, some negative results might be due to the limited sample size of these investigations.

In spite of the above limitations, our study does support the possible advantages offered by antihypertensive treatment based on a CCB or on its combination with an ACEI in reducing not only BP levels, but also short-term BPV. These findings, and their possible impact on cardiovascular prognosis, pave thus the way for additional longitudinal intervention studies of adequate size, which are needed to definitely clarify these issues. In particular, they may have practical implications for planning larger intervention trials targeting BPV and for improving cardiovascular risk reduction in hypertensive patients of different age. Finally, the methodological information provided by our study on different indices quantifying short-term 24 h BPV might help in achieving a better standardization of BPV assessment in clinical trials.

## Methods

### Subjects and studies design

We considered ABPM data recorded in two previous cross-over studies that involved 1) middle-age hypertensive patients receiving monotherapy with an ACEI and a CCB (Study 1), and 2) elderly hypertensive patients treated with the same ACEI and the same CCB as monotherapy or in combination (Study 2), see Fig. 1. Details are reported hereafter.

Both study protocols were approved by the local Ethics Committee of the centers involved (TUKIJA – Lääketieteellinen tutkimuseettinen jaosto, Helsinki, Finland; HUS – Sisatautien eettinen komitea HYKS Meilahden Sairaala, Helsinki, Finland; Comité Ético de Investigación Clínica, Santiago de Compostela, Spain; Comité Ético de Investigación Clínica, Madrid, Spain) and were conducted in accordance with the Declaration of Helsinki and Good Clinical Practice. All subjects gave written informed consent to participate.

### Study 1 (middle-age patients)

This was a single-blind cross-over study conducted on 64 patients with essential hypertension (27 females, 37 males; age 52.9 ± 9.5 yrs, mean ± SD), that received the calcium antagonist Lercanidipine (Zanidip, Recordati, Italy) and the ACEI Enalapril (Renitec, MSD, Belgium) in a random sequential order. Each patient was also randomly assigned to receive L at doses of 10 mg or 20 mg once-a-day, and E at doses of 10 mg or 20 mg once-a-day.

Each treatment lasted 8 weeks and was preceded by 3 weeks of wash out. Initial E10 mg and L10 mg doses could be increased to E20 mg and L20 mg, if clinic DBP remained higher than 90 mmHg or decreased by less than 10 mmHg after one month of treatment. Each subject underwent a 24-hour ABPM immediately before and after each treatment. The ABPM device was set to obtain readings of systolic SBP, DBP and MAP every 15 minutes from 6 am to 10 pm and every 30 minutes from 10 pm to 6 am.

### Study 2 (elderly patients)

This was a four-way, balanced, crossover study conducted on elderly hypertensive patients. Inclusion criteria were: age between 60 and 85 years, baseline clinic SBP between 160 and 179 mmHg and clinic DBP lower than 110 mmHg in sitting position, combined with a mean daytime SBP ≥ 135 mmHg at baseline, measured by ABPM. Patients with history of cerebrovascular or cardiac complications were excluded.

After a wash-out period of 2 weeks, each patient was randomized according to a double-blind design to receive for 4 weeks placebo, L10, E20, and the combination of the two active treatments (L10 + E20) once/day, all administered according to randomized sequences, for a total therapy duration of 16 weeks. At the end of each treatment, patients performed a 24-hour ABPM. As in study 1, the ABPM device was set to obtain readings every 15 minutes from 6 am to 10 pm and every 30 minutes from 10 pm to 6 am. Seventy-five patients were randomized (see^20^ for details). In the present study we considered the 66 of them (31 females, 35 males; age 65.5 + 4.7 yrs) that completed at least three treatments with a valid ABPM recording after each treatment.

For both studies, 24-hour ABPM recordings were performed with a validate device applied to the non-dominant arm (Spacelabs Healthcare, Issaquah, WA).

### Short-term BPV Indices

For each recording, mean values of SBP, MAP and DBP were computed over the whole 24-hour tracing. Two indices of short-term BPV were estimated: the weighted 24 h standard deviation and the Average Real Variability.

Night-time sleep period (SLEEP) and day-time wake period (WAKE) were identified based on the “wake up” and “go to bed” times that patients reported in their diary, and SD_W_ was calculated for SBP, MAP and DBP separately as:

1 $${{\rm{SD}}}_{{\rm{W}}}=({{\rm{n}}}_{{\rm{SLEEP}}}\times {{\rm{SD}}}_{{\rm{SLEEP}}}+{{\rm{n}}}_{{\rm{WAKE}}}\times {{\rm{SD}}}_{{\rm{WAKE}}})/({{\rm{n}}}_{{\rm{SLEEP}}}+{{\rm{n}}}_{{\rm{WAKE}}})$$

where n_SLEEP_ and n_WAKE_ are the number of ABPM measures for each BP series during SLEEP and WAKE periods, SD_SLEEP_ and SD_WAKE_ are the corresponding standard deviations^10^.

The Average Real Variability (ARV) was calculated as:

2 $${\rm{AVR}}=\frac{\sum _{k=1}^{n-1}{w}_{k}\times |B{P}_{k+1}-B{P}_{k}|}{\sum _{k=1}^{n-1}{w}_{k}}$$

with ${w}_{k}={t}_{k+1}-{t}_{k}$ being the time interval between the *k* + 1 and *k* consecutive measures, and *n* the total number of BP measures (SBP, MAP or DBP) in 24 hours^8^.

The SD_W_ and ARV indices offer a somewhat different quantification of short-term BPV. SD_W_ is a measure of BP dispersion around the mean, like the traditional standard deviation; however, at variance from the conventional 24-hour standard deviation, it removes the difference between day and night BP levels from the measure of BP values dispersion. By contrast, ARV is not a measure of dispersion of BP values, but a measure of dispersion of the differences between consecutive BP readings. The paragraph “SD_W_ vs. ARV” illustrates how SD_W_ and ARV weight differently faster and slower components of short-term BPV.

### Statistical Analysis

Normality of distributions of BP mean values and of short-term BPV indices was tested in each condition and for each treatment separately by the Shapiro-Wilks test. Short-term BPV indices passed the normality test after log-transformation. Differences between BEFORE and AFTER conditions for the four treatments (L10, L20, E10 and E20) of study 1 and comparison among the four treatments (placebo, L10, E20 and L10 + E20) of study 2 were performed by applying Linear Mixed-Effects Models with a posteriori contrasts accounting for repeated measurements and compound symmetry covariance structure, fitting the models by maximizing the restricted log-likelihood. For multiple post-hoc comparisons, the expected rate of false-positive results for all positive results was controlled by the false discovery rate algorithm^21^.

For some theoretical probability distributions (like the exponential distribution), mean and standard deviation are not independent quantities, but any decrease (or increase) of the mean is mathematically associated with a decrease (or increase) of standard deviation. To exclude that a similar mechanism, possibly caused by a particular data distribution, may be responsible for observing a reduction of BPV after a treatment that reduced the mean BP, we evaluated the associations between decreases in 24 hour BP mean and short-term standard deviation. This was done by calculating differences in the 24 hour BP mean (ΔMEAN_24H_) and in SD_W_ of BP(ΔSD_W_) between the BEFORE-treatment and the AFTER-treatment conditions (study 1) or between placebo and each active treatment (study 2). Then, linear regressions between ΔMEAN_24H_ and ΔSD_W_ were calculated for each treatment. An association between decreases in mean and in standard deviation is revealed by a statistically significant correlation and a positive slope (β_1_ coefficient). The threshold for statistical significance was set at 0.05; all analyses were performed using R Core Team software (2015), Vienna, Austria.

### SD_W_ vs. ARV

ARV and SD_W_ indices assess the effects of treatments on short-term BPV with different sensitivity. The reason lies in the different nature of the variability components they quantify. SD_W_ measures the standard deviation of BP residuals after removal of nighttime and daytime levels, quantifying all the variability components of BP residuals with the same weight, regardless of their oscillation periods. By contrast, ARV measures the amplitude of the difference between consecutive BP samples, in this way giving a greater weight to the faster components.

In fact, let’s consider a hypothetical sinusoidal component, *BP*_*i*_*(t)*, of amplitude *A*_*i*_ and period *T*_*i*_:

3 $$B{P}_{i}(t)={A}_{i}\,\sin \,\frac{2\pi t}{{T}_{i}}$$

and assume that BP is measured every Δ*t* seconds, so that the *k*-th sample corresponds to the time instant *t*_*k*_ = *k*Δ*t* seconds. The difference between two successive samples, *t*_*k+1*_ and *t*_*k*_, is:

4 $$B{P}_{i}({t}_{k+1})-B{P}_{i}({t}_{k})={A}_{i}\,\sin (\frac{2\pi ({t}_{k}+{\rm{\Delta }}t)}{{T}_{i}})-{A}_{i}\,\sin (\frac{2\pi {t}_{k}}{{T}_{i}})$$

By applying the sum-to-product trigonometric identity, Equation (4) can be rewritten as:

5 $$B{P}_{i}({t}_{k+1})-B{P}_{i}({t}_{k})={D}_{i}\,\sin (2\pi \frac{{t}_{k}}{{T}_{i}}+\phi )$$

with

6 $${D}_{i}=2{A}_{i}\,\sin (\pi \frac{{\rm{\Delta }}t}{{T}_{i}})$$

and

7 $$\phi =\pi \frac{{\rm{\Delta }}t}{{T}_{i}}+\frac{\pi }{2}$$

Therefore also the series of differences between consecutive BP samples is a sinusoid with period *T*_*i*_, as *BP*_*i*_*(t)*, but with different phase and different amplitude. In particular, the amplitude depends on the Δ*t*/*T*_*i*_ ratio, and lower is this ratio, lower is *D*_*i*_. This means that BP oscillations with different period contribute differently to ARV.

**Figure 4:**
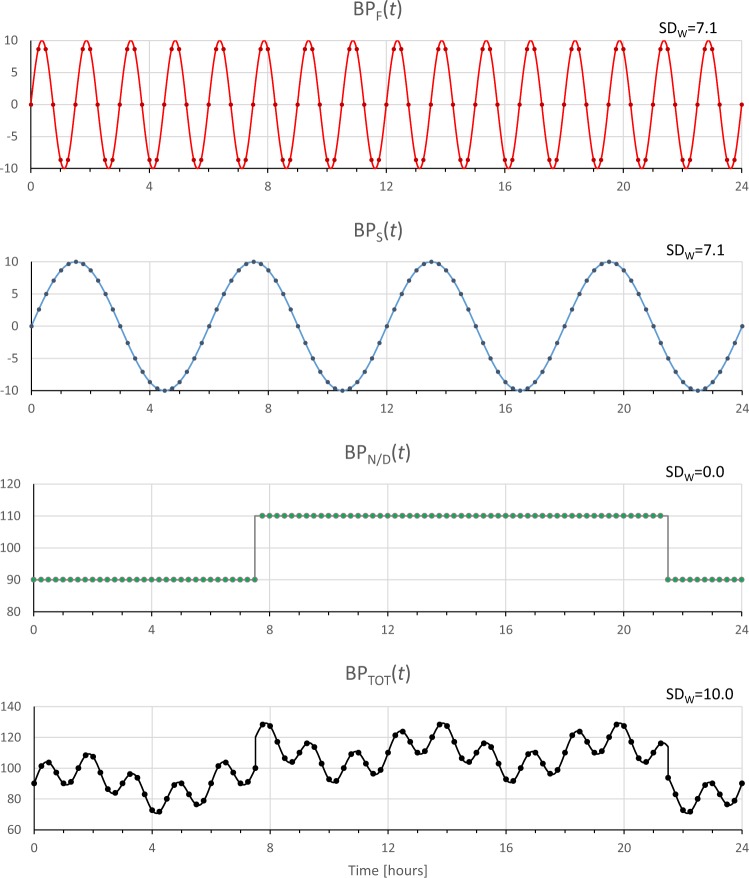
BP_TOT_ (*lower panel*) is a synthesized BP continuous signal obtained by summing a fast (BP_F_) and a slow (BP_S_) sinusoidal oscillation to a night/day modulation with square-wave shape (BP_N/D_). The dot symbols indicate simulated measures obtained with an ABPM device that samples the BP signals every 15 minutes; SD_W_ is the weighted standard deviation of each sampled BP series.

Discrepancies between SD_W_ and ARV can be understood simulating a *BP(t)* series sampled every 15 minutes as sum of three components (Fig. 4). First component, BP_F_, is a fast sinusoidal oscillation with 10-mmHg amplitude and 90-minute period (*T*_*F*_ = 3600 s). Second component, BP_S_, is a slow sinusoidal oscillation with the same amplitude (10 mmHg) and 5-hour period (*T*_*S*_ = 14400 s). Third component, BP_N/D_, represents the night/day modulation by a square wave between 110 mmHg during daytime (from 7:30 to 21:30) and 90 mmHg during nighttime (from 21:30 to 7:30). The SD_W_ of the night/day modulation is clearly null. The fast and slow oscillations have the same SD_W_ (7.1 mmHg) because SD_W_ depends on the amplitude and not on the period of the oscillation. The SD_W_ of the resulting simulated BP series, BP_TOT_, is 10.0 mmHg, value that coincides with the square root of the sum of SD_W_’s squared of the three components (we recall that the power of the sum of sinusoids with different frequencies equals the sum of the power of each sinusoid). Therefore, fast and slow oscillations contribute equally to the SD_W_ of BP_TOT_.

**Figure 5:**
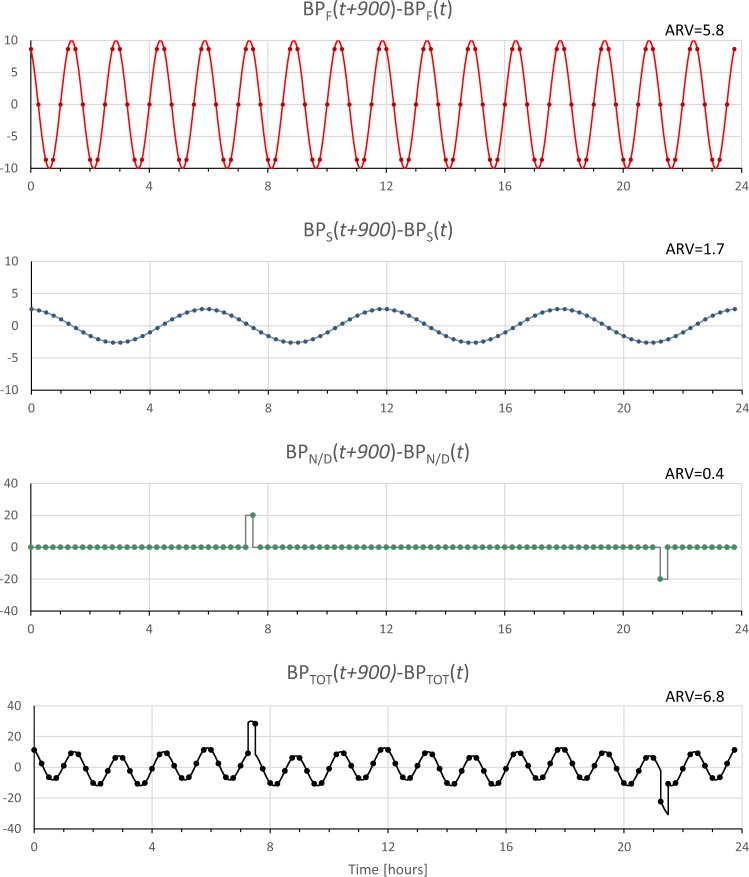
Synthesized BP signals of Fig. 4 after differentiation; ARV is the average real variability of time series obtained by simulating the sampling of an ABPM device every 15 minutes.

This is not the case for ARV. To evaluate ARV, we should first calculate the difference between consecutive BP samples. By applying Equation (4), with Δ*t*/*T*_*F*_ = 900/5400 and Δ*t*/*T*_*S*_ = 900/21600, we obtain that the amplitude of the differentiated fast component is *D*_*F*_ = 10.0 and of the differentiated slow component is *D*_*S*_ = 2.6. Figure 5 shows clearly the lower amplitude of the slow oscillation, and therefore ARV is higher for the faster oscillation. The differentiated night/day component is zero at all times except two instants with the fast transition from night to day and from day to night, which contribute minimally to the overall ARV.

Table 2 quantifies how the fast and the slow components contribute to the overall short term variability. It compares SD_W_ and ARV of BP_TOT_, considered as the 100% reference, with the values estimated by excluding from BP_TOT_ only the fast components (BP_TOT_-BP_FAST_), or only the slow component (BP_TOT_-BP_SLOW_). Table 2 points out that the contribution to ARV of BP_TOT_ of the slow component is much smaller than the contribution of the fast component, while these two components contribute similarly to the SD_W_ of BP_TOT_.

**Table 2:** Indices of short-term BPV evaluated on simulated BP_TOT_ series of Fig. 4 after removing the fast component only (BP_F_) or the slow component only (BP_S_), expressed as percentage of the reference value.

	BP_TOT_	BP_TOT_-BP_F_	BP_TOT_-BP_S_
SD_W_	100%	71%	71%
ARV	100%	31%	92%

Considering the frequency of readings of the ABPM devices used in study 1 and 2, the slow components that affect SD_W_ without influencing ARV importantly are those with time scales of hours. These could be the BP changes associated with the circulatory long-term control of the renin-angiotensin-aldosterone system over the 24-hour period and, during sleep, the possible long-term BP modulations associated with the 100-minute alternation of REM and NREM sleep. By contrast, the faster BP changes that are expected to influence ARV more than SD_W_ could be the BP fluctuations associated with sleep related breathing disorders during night-time, or the BP changes associated with postural changes or with everyday activities during daytime.
